# Supplementary material for: A single regulator NrtR controls bacterial NAD+ homeostasis via its acetylation
Source: eLife. 2019 Oct 9;8:e51603. doi: 10.7554/eLife.51603 (PMC6800001; doi:10.7554/eLife.51603)
Supplement: Supplementary file 1. [file elife-51603-supp1.doc]

**Supplementary File 1** Strains and plasmids used in this study

| Strains and plasmids | Relevant characteristics | Origins |
| --- | --- | --- |
| Strains |  |  |
| FYJ500 | *E. coli* DH5α, a cloning host | Lab stock |
| FYJ501 | *E. coli* BL21 (DE3), a protein expression host | Lab stock |
| FYJ594 | Bl21(DE3) with pET28a-*nrtR* | This work |
| FYJ1001 | Bl21(DE3) with pET28a-*nrtR*-D167A | This work |
| FYJ1002 | Bl21(DE3) with pET28a-*nrtR*-T169A | This work |
| FYJ1003 | Bl21(DE3) with pET28a-*nrtR*-N170A | This work |
| FYJ1005 | Bl21(DE3) with pET28a-*nrtR*-R173A | This work |
| FYJ1006 | Bl21(DE3) with pET28a-*nrtR*-R196A | This work |
| FYJ1007 | Bl21(DE3) with pET28a-*nrtR*-K179A | This work |
| FYJ1008 | Bl21(DE3) with pET28a-*nrtR*-K134A | This work |
| FYJ1010 | Bl21(DE3) with pET28a-*nrtR*-K134R | This work |
| FYJ1009 | Bl21(DE3) with pET28a-*nrtR*-K134Q | This work |
| FYJ1117 | Bl21(DE3) with pET28a-*nrtR*-Q54E&K58E&D60G | This work |
| FYJ673 | DH5α with pMV261-*null*-*lacZ* | 1 |
| FYJ674 | DH5α with pMV261-*hsp60*-lacZ | 1 |
| FYJ675 | DH5α with pMV261-*nrtR*p-*lacZ* | This work |
| FYJ665 | *M. smegmatis* MC^2^155 with pMV261-*null*-*lacZ* | 1 |
| FYJ666 | Δ*nrtR*(MSMEG_3198) with pMV261-*null*-*lacZ* | This work |
| FYJ667 | *M. smegmatis* MC^2^155 with pMV261-*hsp60*-*lacZ* | 1 |
| FYJ668 | Δ*nrtR*(MSMEG_3198) with pMV261-*hsp60*-*lacZ* | This work |
| FYJ669 | *M. smegmatis* MC^2^155 with pMV261-*nrtRp*-*lacZ* | This work |
| FYJ670 | Δ*nrtR*(MSMEG_3198) with  pMV261- *nrtRp*-*lacZ* | This work |
| FYJ563 | *M. smegmatis* MC^2^155 | Lab stock |
| FYJ1080 | *nrtR* (MSMEG_3198) in frame deletion strain of *M. smegmatis* | This work |
| FYJ1082 | Δ*nrtR* complementary strain of *M. smegmatis* | This work |
| FYJ1090 | Δ*pat* (MSMEG_5458) mutant of *M. smegmatis* MC2155 | This work |
| FYJ1088 | Δ*cobB* (MSMEG_5175) mutant of *M. smegmatis* MC^2^155 | This work |
| FYJ1101 | Δ*pta* (MSMEG_0783) mutant of *M. smegmatis* MC^2^155 | This work |
| FYJ1102 | Δ*ackA* (MSMEG_0784) mutant of *M. smegmatis* MC^2^155 | This work |
| FYJ1103 | Δ*pta+*Δ*ackA* double mutant of *M. smegmatis* MC^2^155 | This work |
| FYJ1011 | *M. smegmatis* MC^2^155 with pMV261-*nrtR*-6×his | This work |
| FYJ1100 | Δ*pat* (MSMEG_5458) mutant with pMV261-*nrtR*-6×his | This work |
| FYJ1097 | Δ*cobB* (MSMEG_5175) mutant with pMV261-*nrtR*-6×his | This work |
| FYJ1094 | *M. smegmatis* MC^2^155 with pMV261-*usp*-6×his | This work |
| FYJ1095 | Δ*cobB* (MSMEG_5175) mutant with pMV261-*usp*-6×his | This work |
| FYJ1096 | Δ*pat* (MSMEG_5458) mutant with pMV261-*usp*-6×his | This work |
| FYJ1086 | *M. smegmatis* MC^2^155 NrtR(K134A) chromosome mutation | This work |
| FYJ1084 | *M. smegmatis* MC^2^155 NrtR(K134R) chromosome mutation | This work |
| FYJ1087 | *M. smegmatis* MC^2^155 NrtR(K134Q) chromosome mutation | This work |
| FYJ1540 | NrtR(K134A) chromosome mutation with pMV261-*nrtRp*-*lacZ* | This work |
| FYJ1541 | *NrtR(K134R) chromosome mutation* with pMV261-*nrtRp*-*lacZ* | This work |
| FYJ1542 | NrtR(K134Q) chromosome mutation with pMV261-*nrtRp*-*lacZ* | This work |
| Plasmids |  |  |
| pET28a | T7-driven expression vector, Km^R^ | Lab stock |
| pET28a-*nrtR* | *nrtR* in BamHI and XhoI sites of pET28a | This work |
| pET28a-*nrtR*-D167A | *nrtR* (D167A) in BamHI and XhoI sites of pET28a | This work |
| pET28a-*nrtR*-T169A | *nrtR* (T169A) in BamHI and XhoI sites of pET28a | This work |
| pET28a-*nrtR*-N170A | *nrtR* (N170A) in BamHI and XhoI sites of pET28a | This work |
| pET28a-*nrtR*- R173A | *nrtR* (R173A) in BamHI and XhoI sites of pET28a | This work |
| pET28a-*nrtR*- R196A | *nrtR* (R196A) in BamHI and XhoI sites of pET28a | This work |
| pET28a-*nrtR*-K179A | *nrtR* (K179A) in BamHI and XhoI sites of pET28a | This work |
| pET28a-*nrtR*-K134A | *nrtR* (K134A) in BamHI and XhoI sites of pET28a | This work |
| pET28a-*nrtR*-K134R | *nrtR* (K134R) in BamHI and XhoI sites of pET28a | This work |
| pET28a-*nrtR*-K134Q | *nrtR* (K134Q) in BamHI and XhoI sites of pET28a | This work |
| pMV261 | *ColE1* replicon, pAL5000 replicon, *hsp60* promoter, expression vector | 2 |
| pMV261-*nrtR*-6×his | *nrtR*-6×his in BamHI and SalI sites of pMV261 | This work |
| pMV261-*usp*-6×his | *usp* (MSMEG­_4207)*-*6×his in HindIII and NheI sites of pMV261 | This work |
| pMV261-*null*-*lacZ* | *lacZ* in XbaI and NheI sites of pMV261 | 1 |
| pMV261-*hsp60*-*lacZ* | *lacZ* in HindIII and NheI sites of pMV261 | 1 |
| pMV261-*nrtR*p-*lacZ* | *lacZ* with the promoter of *nrtR* in XbaI and NheI sites of pMV261 | This work |
| pMV261-*nadABC*p-*lacZ* | *lacZ* with the promoter of *nadABC* in XbaI and NheI sites of pMV261 | This work |
| pMind | pAL5000 replicon, *ColE1* replicon, used for gene-knockout, Kan^R^, Hyg^R^ | 3 |
| pGoAL17 | pBR322 replicon, used for gene-knockout, Amp^R^ | 4 |
| pMind-*nrtR*UD | Intermediate vector in *nrtR*-knockout experiments | This work |
| pMind-*nrtR*UD-*sacB*-*lacZ* | Suicide plasmid used for *nrtR*-knockout | This work |
| pMind-*pat*UD | Intermediate vector in *pat*-knockout experiments | This work |
| pMind-*pat*UD-*sacB*-*lacZ* | Suicide plasmid used for *pat* -knockout | This work |
| pMind-*cobB*UD | An intermediate vector for *cobB*-knockout | This work |
| pMind-*cobB*UD-*sacB*-  *lacZ* | Suicide plasmid used for *cobB* -knockout | This work |
| pMind-*pta*UD-*sacB*-  *lacZ* | Suicide plasmid used for *pta* -knockout | This work |
| pMind-*ackA*UD-*sacB*-  *lacZ* | Suicide plasmid used for *ackA* -knockout | This work |
| pMind-*ackA*U’D’-*sacB*-  *lacZ* | Suicide plasmid used for *pta*+*ackA* -knockout | This work |

Supplementary references

1. Tang Q, Li X, Zou T, Zhang H, Wang Y, Gao R*, et al.* *Mycobacterium smegmatis* BioQ defines a new regulatory network for biotin metabolism. *Mol Microbiol* 2014, **94**(5)**:** 1006-1023.

2. Yang M, Gao C, Cui T, An J, He Z-G. A TetR-like regulator broadly affects the expressions of diverse genes in *Mycobacterium smegmatis*. *Nucleic Acids Res* 2012, **40**(3)**:** 1009-1020.

3. Parish T, Stoker NG. Use of a flexible cassette method to generate a double unmarked *Mycobacterium tuberculosis tlyA plcABC* mutant by gene replacement. *Microbiology* 2000, **146**(8)**:** 1969-1975.

4. Blokpoel MCJ, Murphy HN, O'Toole R, Wiles S, Runn ESC, Stewart GR*, et al.* Tetracycline-inducible gene regulation in mycobacteria. *Nucleic Acids Res* 2005, **33**(2)**:** e22-e22.
