# Supplementary material for: A single regulator NrtR controls bacterial NAD+ homeostasis via its acetylation
Source: eLife. 2019 Oct 9;8:e51603. doi: 10.7554/eLife.51603 (PMC6800001; doi:10.7554/eLife.51603)
Supplement: Supplementary file 2. [file elife-51603-supp2.doc]

**Supplementary File 2** Primers used in this study

| Primers | Primer sequences |
| --- | --- |
| *nrtRp* (XbaI)-F | 5’-GC*T CTA GA*T CGG CCG CGA TGC GGG AC-3’ |
| *nrtRp* (HindIII)-R | 5’-CCC *AAG CTT* CAC TTC GTG GGC GGT GCT A-3’ |
| *nadp* (XbaI)-F | 5’-GC*T CTA GA*G GAT CGG AGA ACA CCG CGA-3’ |
| *nadp* (HindIII)-R | 5’-CCC *AAG CTT* CAC GGT GGC CTC CTC ATC GA-3’ |
| *lacZ* (HindIII)-F | 5’-CCC *AAG CTT* ATG GAT CCC GTC GTT TTA CA-3’ |
| *lacZ* (NheI)-R | 5’-CTA *GCT AGC* TTA TTT TTG ACA CCA GAC CAA C-3’ |
| *nrtR* (BamHI)-F | 5’-CG*G GAT CC*A GTG CTC GCC GTC GTG TT-3’ |
| *nrtR* (SalI)-R | 5’-ACG C*GT CGA C*TC AGT GGT GGT GGT GGT GGT GCC CGG GCG GTC GCA ACG-3’ |
| *nrtR* (BamHI)-F | 5’-CG*G GAT CC*G TGC TCG CCG TCG TGT T-3’ |
| *nrtR* (XhoI)-R | 5’-CCG *CTC GAG* TCA CCC GGG CGG TCG CAA-3’ |
| *nrtR*-D167A-F | 5’-TTG GAC TAC CAG GTG GCC GCC ACC AAC CTG CAG CG-3’ |
| *nrtR*-D167A-R | 5’-GCC ACC TGG TAG TCC AAC GCG GCG CTG TAG AC-3’ |
| *nrtR*-T169A-F | 5’-TGG ACG CCG CCA ACC TGC AGC GGG TTC TCG AA-3’ |
| *nrtR*-T169A-R | 5’-CAG GTT GGC GGC GTC CAC CTG GTA GTC CAA CG-3’ |
| *nrtR*-N170A-F | 5’-ACC GCC CTG CAG CGG GTT CTC GAA CGC CGC AAG-3’ |
| *nrtR*-N170A-R | 5’-AAC CCG CTG CAG GGC GGT GGC GTC CAC CTG GTA G-3’ |
| *nrtR*-R173A-F | 5’-AAC CTG CAG GCG GTT CTC GAA CGC CGC AAG GTG-3’ |
| *nrtR*-R173A-R | 5’-GAG AAC CGC CTG CAG GTT GGT GGC GTC CAC CTG-3’ |
| *nrtR-*K179A-F | 5’-AAC GCC GCG CGG TGA TCA CCC GCA CCG GGA CCA-3’ |
| *nrtR-*K179A-R | 5’-TGA TCA CCG CGC GGC GTT CGA GAA CCC GCT GCA-3’ |
| *nrtR-*R196A-F | 5’-GCG CCG GCC GCC CTG TTC CGG TTC ACC GAG TC-3’ |
| *nrtR-*R196A-R | 5’-AAC AGG GCG GCC GGC GCC CCG CCG CTG CGG CCC GA-3’ |
| *nrtR* (Q54E&K58E&D60G)-F | 5’-AAC TCG CCG AGG AAG TGG GAC TTC GCG AGC TCG CCC AC-3’ |
| *nrtR* (Q54E&K58E&D60G)-R | 5’-CAC TTC CTC GGC GAG TTC ACG CCG CAC CGA CGT GGT-3’ |
| *nrtR*-K134Q-F | 5’-TGG TCG CCC AGC TTT CCT A-3’ |
| *nrtR-*K134Q-R | 5’-TAG GAA AGC TGG GCG ACC A-3’ |
| *nrtR-*K134R-F | 5’-TGG TCG CCA GGC TTT CCT A-3’ |
| *nrtR-*K134R-R | 5’-TAG GAA AGC CTG GCG ACC A-3’ |
| *nrtR-*K134A-F | 5’-TGG TCG CCG CGC TTT CCT A-3’ |
| *nrtR-*K134A-R | 5’-TAG GAA AGC GCG GCG ACC A-3’ |
| *nrtR*-U (PacI)-F | 5’-CC*T TAA TTA A*GT GAT CAT CTT CAT GTA CGT G-3’ |
| *nrtR*-U-overlap-R | 5’-AGC CGA CTC ACA CTT CGT GGG CGG TGC T-3’ |
| *nrtR-*D-overlap-F | 5’-CCA CGA AGT GTG AGT CGG CTG GAC TCT TAG-3’ |
| *nrtR*-D-(NheI)-R | 5’-CTA *GCT AGC* TGT CGA GGA TCT GTT CGA GC-3’ |
| *usp* (HindIII)-F | 5’-CCC *AAG CTT* ATG TGA TCG TGG TCG GTT ACA-3’ |
| *usp* (NheI)-R | 5’-CTA *GCT AGC* TCA GTG GTG GTG GTG GTG GTG GAA CCC ATG TGG CTT GAC C-3’ |
| *pat-*U-(PacI)-F | 5’-CC*T TAA TTA A*TG CGG TCA GCA GTG AGT TGA-3’ |
| *pat-*U-R | 5’-GCC CCA CTC ACA CAT TCC CAG GCT ACG ACG-3’ |
| *pat-*D-F | 5’-TGG GAA TGT GTG AGT GGG GCG GAC AAC AG-3’ |
| *pat-*D-(NheI)-R | 5’-CTA *GCT AGC* CGG TTG ATC GCC CAG AAG AC-3’ |
| *cobB*-U-(PacI)-F | 5’-CC*T TAA TTA A*CC AGC TTC TAC CGC CTC AA-3’ |
| *cobB*-U-R | 5’-CTA GAG TTC ACA CGT CAA CCA AGG TAG CGG-3’ |
| *cobB*-D-R | 5’-GGT TGA CGT GTG AAC TCT AGG GCC GCA CC-3’ |
| *cobB-*D-(NheI)-R | 5’-CTA *GCT AGC* CCC GAG CTG TAC CGG CTG-3’ |
| *pta-*U-(PacI)-F | 5’-CCT TAA TTA ACG AGG TCG ACC AGG TGA T-3’ |
| *pta-*U-R | 5’-AGT ACT GTC ACA CGA CCT ACA GCA TCC C-3’ |
| *pta-*D-F | 5’-GTA GGT CGT GTG ACA GTA CTC GTC GTC AAC-3’ |
| *pta-*D-(NheI)-R | 5’-CTA *GCT AGC* TCA TCG ATC TCG ATG CCC A-3’ |
| *ackA-*U-(PacI)-F | 5’-CCT TAA TTA ACG ATC CCC GAG GTC ACG A-3’ |
| *ackA*-U-R | 5’-AAA CGA GCT ACA CGA CCG GCC CTG CG-3’ |
| *ackA*-D-F | 5’-GCC GGT CGT GTA GCT CGT TTT GCG TCG AGT-3’ |
| *ackA-*D-(NheI)-R | 5’-CTA *GCT AGC* AAT TCT GCG GCC ATG ACC G-3’ |
| *pta+ackA-*U’-(PacI)-F | 5’-CC*T TAA TTA A*CG AGG TCG ACC AGG TGA T-3’ |
| *pta+ackA-*U’-R | 5’-AAA CGA GCT ACA CAC GAC CTA CAG CAT CC-3’ |
| *pta+ackA-*D’-F | 5’-GTA GGT CGT GTG TAG CTC GTT TTG CGT CGA G-3’ |
| *pta+ackA-*D’-(NheI)-R | 5’-CTA *GCT AGC* AAT TCT GCG GCC ATG ACC G-3’ |
| *nrtR-*probe-F  (57 bp) | 5’-CTC CTC ATC GAG TTC AG**G TTT TCG ACT TAT AAT CGA AAA C**AT GCC CAA TCG TAG CAC-3’ |
| *nrtR-*probe-R  (57 bp) | 5’-GTG CTA CGA TTG GGC AT**G TTT TCG ATT ATA AGT CGA AAA C**CT GAA CTC GAT GAG GAG-3’ |
| *vprA*-probe-F  (56 bp) | 5’-CGG TAG CTG **ACA ATG C**CA T**AC AAT** **TC**T GCG ACA GAT TTG TTA CAG AGA GAG GGT TA-3’ |
| *vprA*-probe-R  (56 bp) | 5’-TAA CCC TCT CTC TGT AAC AAA TCT GTC GCA **GAA TTG T**ATG**GC ATT GT**C AGC TAC CG-3’ |
| 1-F | 5’-ACCTGGAACACGACGGCGAGC-3’ |
| 1-R | 5’-GTCCACAATCCGGTCCGCGAG-3’ |
| 2-F | 5’-CGACGCTGCTGGCGCACAACTAC-3’ |
| 2-R | 5’-ACCGTCTTGTCGGGGCTGAGGAT-3’ |
| 3-F | 5’-GTGCACGTGGACCCGGAGACC-3’ |
| 3-R | 5’-CGTCGGTTCGTTGCTGCCAGT-3’ |
| 4-F | 5’-GGCTCGGCTGACGACACCA-3’ |
| 4-R | 5’-CGCGGATTCGTCGAACAGG-3’ |
| 5-F | 5’-TCGATGACAGTCCGTATCCA-3’ |
| 5-R | 5’-ACCGTAGGCGAGGTCTTCTT-3’ |
| 6-F | 5’-TCAAGGCCGTCCGCGAAGAGG-3’ |
| 6-R | 5’-TCAGCCCGCCCGAAGACTCCA-3’ |
| 7-F | 5’-CCTGGACTTCTGAGGCGGTTG-3’ |
| 7-R | 5’-AGAGCAGCGACACCAAGAGCG-3’ |
| CK16S-F | 5’-TAAGTCCCGCAACGAGCGCAACC-3’ |
| CK16S-R | 5’-TCACGGCATCACAGCCCTTTGTA-3’ |

* The sites of restriction enzymes are underlined letters in italic, and the NrtR-binding palindromes are indicted with bold letters.
