# Supplementary material for: A single regulator NrtR controls bacterial NAD+ homeostasis via its acetylation
Source: eLife. 2019 Oct 9;8:e51603. doi: 10.7554/eLife.51603 (PMC6800001; doi:10.7554/eLife.51603)
Supplement: Supplementary file 3. [file elife-51603-supp3.doc]

**Supplementary File 3** Nudix family protein in *M. smegmatis* MC^2^ 155

| Gene | Annotation | Nudix motif (G[X5]E[X7]REUXEEXGU) | Length (aa) | Acc. no. |
| --- | --- | --- | --- | --- |
| MSMEG0197 | Nudix hydrolase | GWCDVLETPAQAVAKEVREEAGLI | 214 | YP_884613 |
| MSMEG1268 | ADP-ribose pyrophosphatase (YjhB) | TFLHQGETLAEAVNRALSTKANVQGLRPRQ | 222 | YP_885658 |
| MSMEI3116 | ADP-ribose pyrophosphatase (YjhB) | GRLRDDEDLTTSVRRQLAEKVDLRELAHLEQ | 230 | AFP39580 |
| MSMEI2862 | Nudix hydrolase | GAVQKGEDLAETAARELFEETGLRIEPSALVG | 351 | AFP39326 |
| MSMEI1785 | Nudix hydrolase | GGHCEDTDPDIHAAALREATEESGIEGLTIDPDLAAL | 182 | AFP38257 |
| MSMEG5118 | ADP-ribose pyrophosphatase (YjhB) | GTAPDRQDLEPHALAERELREETGLRAAHLVK | 179 | YP_889364 |
| MSMEG6617 | Nudix hydrolase, Coenzyme A pyrophosphatase (CoAse) | GRVDPGETVEEAALRELDEELGVRLPESTVL | 239 | YP_890828 |
| LJ0034200 | Nudix hydrolase, Ap6A_hydrolase | GHIELGETAEQTAIREVAEETGIQGSVLAA | 304 | AIU11878 |
| LJ0011885 | Nudix hydrolase, Diadenosine hexaphosphate (Ap6A_hydrolase) | GKLDQGETEPVAAAREIHEETGHTAVLGRRLG | 311 | AIU07573 |
| MSMEG1047 | Nudix hydrolase |  | 273 | YP_885444 |
| MSMEG2390 | Nudix family, Ap6A_hydrolase | GKLDQGETEPVAAAREIHEETGHTAVLGRRLG | 322 | YP_886730 |
| MSMEG3198 | ADP-ribose pyrophosphatase (YjhB) | GRLRDDEDLTTSVRRQLAEKVDLRELAHLEQ | 221 | YP_887512 (NrtR) |
| MSMEG4488 | Nudix family, hydrolase | GKIEPGEQPLAALHREVVEELGTAVDPTSV | 134 | YP_888760 |
| MSMEG0790 | Nudix family hydrolase | GARDSHETPEQAAVREAHEEAGLPAEQL | 159 | YP_885194 |
| MSMEG2936 | Nudix family hydrolase, LemA family | GAVQKGEDLAETAARELFEETGLRIEPSALVG | 351 | YP_887255 |
| MSMEG1827 | Nudix family hydrolase | GHCEDTDPDIHAAALREATEESGIEGLTIDPDLAAL | 182 | YP_886198 |
| MSMEI1410 | Nudix family hydrolase | GGVLDPGEAPLQAATRELHEELGITGLIPTPL | 184 | AFP37883 |
| MSMEI2330 | Hydrolase MutT1, Ap6A_hydrolase | GKLDQGETEPVAAAREIHEETGHTAVLGRRL | 321 | AFP38798 |
| MSMEI5016 | MutT2/NUDIX hydrolase, putative mutator protein | GKVTPGESDADALARELREELGVDVAVGE | 130 | AFP41460 |
| MSMEG6927 | MutT/nudix family protein, Ap6A_hydrolase | GHIELGETAEQTAIREVAEETGIQGSVLAA | 297 | YP_891121 |
| MSMEG1946 | NADH pyrophosphatase, Nudix-like super family |  | 311 | YP_886312 |
| MSMEI6737 | MutT/NUDIX family protein, Ap6A_hydrolase | GHIELGETAEQTAIREVAEETGIQGSVLAA | 311 | AFP43163 |
| MSMEG3679 | Phosphohydrolase, Nudix Hydrolase | GEYTDDEDRWAAAQREFAEELGSPPPDGPRREL | 164 | YP_887982 |
| MSMEG1446 | NTP pyrophosphohydrolase, Nudix Hydrolase | GGVLDPGEAPLQAATRELHEELGITGLIPTPL | 173 | YP_885829 |
| MSMEI3592 | hypothetical protein | GEYTDDEDRWAAAQREFAEELGSPPPDGPRREL | 161 | AFP40055 |
| MSMEI0773 | Nudix_Hydrolase, putative mutator protein MutT3 (MutT/nudix family) | GARDSHETPEQAAVREAHEEAGLPAEQLT | 167 | AFP37253 |
| MSMEG3745 | MutT/nudix family protein, DP-ribose pyrophosphatase (ADPRase) | GLLDLGGEPPEVTAARELEEEVGLAASDWRVLV | 206 | YP_888044 |
| MSMEG6185 | NTP pyrophosphohydrolase, Coenzyme A pyrophosphatase (CoAse), a member of the Nudix hydrolase superfamily | GATDPGDTGPVATAFREATEETGVDTSRLH | 262 | YP_890405 |
| MSMEG5148 | CTP pyrophosphohydrolase, MutT_pyrophosphohydrolase | GKVTPGESDADALARELREELGVDVAVGE | 130 | YP_889394 |

* NrtR is marked in red, proteins with conserved Nudix motif signature are marked in blue.
